# Therapeutic effects of stem cell–derived extracellular vesicles in animal models of intervertebral disc degeneration: a systematic review and meta-analysis of species differences and delivery strategies

**DOI:** 10.3389/fbioe.2026.1749916

**Published:** 2026-01-30

**Authors:** Yi-Ping Wei, Yow-Ling Shiue, Chun-Sheng Tsai, Yih-Wen Tarng

**Affiliations:** 1 Department of Orthopedics, Kaohsiung Veterans General Hospital, Kaohsiung City, Taiwan; 2 Institute of Biomedical Sciences, National Sun Yat-sen University, Kaohsiung, Taiwan; 3 Department of Occupational Therapy, Shu-Zen Junior College of Medicine and Management, Kaohsiung City, Taiwan; 4 College of Medicine, National Sun Yat-sen University, Kaohsiung, Taiwan; 5 Office of R&D with International Affairs, Tajen University, Pingtung, Taiwan; 6 Institute of Precision Medicine, National Sun Yat-sen University, Kaohsiung, Taiwan; 7 Department of Orthopedics, Kaohsiung municipal united hospital, Kaohsiung City, Taiwan

**Keywords:** apoptosis, extracellular vesicles, hydrogel delivery system, intervertebral disc degeneration, meta-analysis, rat model, regenerative medicine, stem cell-derived exosomes

## Abstract

**Objectives:**

Intervertebral disc degeneration (IVDD) is a major contributor to chronic low back pain and disability worldwide, yet current treatments remain largely palliative and do not restore disc structure or biomechanical integrity. Stem cell–derived extracellular vesicles (SC-sEVs) have emerged as promising cell-free biologics capable of modulating inflammation, apoptosis, and extracellular matrix homeostasis.

**Methods:**

This systematic review and meta-analysis evaluated the therapeutic efficacy of SC-sEVs in rat models of puncture-induced IVDD, with a specific focus on comparing hydrogel-assisted versus direct (non-hydrogel) delivery strategies. The review was prospectively registered in PROSPERO (CRD420250654980) and conducted according to PRISMA 2020 guidelines.

**Results:**

Comprehensive searches of PubMed, Embase, and the Cochrane Library through August 2025 identified 19 studies enrolling 305 rats. Extracted outcomes included disc height index (DHI), MRI Pfirrmann grade, and histological score. Meta-analysis demonstrated significant improvements in DHI (mean difference [MD] = 12.8%, 95% CI 7.6–18.0), histological grade (MD = −4.1, 95% CI –5.1 to −3.2), and MRI Pfirrmann grade (MD = −1.5, 95% CI –1.8 to −1.2) at 4–8 weeks following treatment. Hydrogel-assisted delivery produced comparable overall efficacy to direct injection but contributed to reduced interstudy heterogeneity. Both human- and rat-derived EVs significantly improved all evaluated outcomes, with human-source EVs showing a modest advantage in MRI grading (P = 0.017). Risk-of-bias assessment indicated generally acceptable methodological quality, and no substantial publication bias was observed.

**Conclusion:**

Overall, SC-sEV therapy demonstrates consistent regenerative benefits in preclinical IVDD models, supporting its translational promise as a minimally immunogenic, cell-free therapeutic for degenerative spine disorders. Future studies employing standardized protocols, mechanistic analyses, and long-term evaluation are needed to facilitate clinical translation.

**Systematic Review Registration:**

https://www.crd.york.ac.uk/PROSPERO/view/CRD420250654980, identifier CRD420250654980.

## Introduction

Intervertebral disc degeneration (IVDD) is a leading cause of chronic low back pain, representing a major source of disability and socioeconomic burden worldwide (Vergroesen et al., 2015; Wu et al., 2020). With the progressive aging of the global population, the prevalence of IVDD continues to rise (Gra et al., 2023) Its pathogenesis is multifactorial, involving mechanical overload, oxidative stress, and age-related changes that disrupt the balance between anabolic and catabolic processes in disc tissues (Silwal et al., 2023). These alterations result in nucleus pulposus cell apoptosis, inflammatory activation, extracellular matrix degradation, and ultimately loss of disc hydration and structural integrity (Costăchescu et al., 2022). Current therapeutic strategies, including conservative treatment and surgical interventions, remain largely palliative and fail to restore disc biology, underscoring the urgent need for regenerative approaches.

Extracellular vesicles (EVs), comprising exosomes and microvesicles, have emerged as promising biologic candidates due to their ability to mediate intercellular communication. By transporting proteins, lipids, and nucleic acids, EVs derived from stem cells can modulate inflammation, regulate matrix metabolism, and reduce apoptosis of disc cells (Gupta et al., 2021; Bhat et al., 2025). Both bone marrow–and adipose-derived stem cell exosomes have demonstrated the capacity to mitigate disc degeneration in experimental settings (Krut et al., 2021). Importantly, xenogeneic stem cell–derived EVs (SC-sEVs) have also shown efficacy in rat models, suggesting that standardized, scalable products may retain cross-species bioactivity (Ambrosio et al., 2024; Jin et al., 2024). However, the specific impact of donor species remains under investigation. While EV structure is evolutionarily conserved, the molecular cargo (e.g., proteins and miRNAs) is species-specific. Therefore, comparing human-derived EVs (xenogeneic) against rat-derived EVs (allogeneic) is essential to confirm that the therapeutic potency of human clinical-grade products is not diminished by species-specific molecular mismatches in preclinical testing.

In addition to the intrinsic therapeutic properties of EVs, their clinical translation may depend on the optimization of delivery strategies. Direct intradiscal injection, while minimally invasive, is often limited by the rapid clearance of EVs from the injection site due to high intradiscal pressure and the leakage of the liquid suspension. Hydrogel-based carriers have attracted attention as intradiscal delivery vehicles, as they can overcome these limitations by providing sustained release and improved retention of exosomes within the avascular disc environment (Zhan et al., 2025; Hu et al., 2024; Shi et al., 2024; Xing et al., 2021). This approach may enhance therapeutic efficacy compared with direct injection of EVs alone.

With the growing number of preclinical studies published in recent years, it has become feasible to focus the evidence synthesis on more specific conditions. Unlike earlier reviews that included multiple species and heterogeneous EV sources, the present systematic review and meta-analysis restricted inclusion to rat models and SC-sEVs interventions (Li et al., 2024). This restriction was applied because rat models offer the most standardized induction protocols (needle puncture) and uniform outcome measures (e.g., DHI, MRI grading), ensuring statistical robustness. In contrast, studies on larger animals (e.g., rabbits, canines) are currently too sparse and heterogeneous in their reporting standards to permit reliable meta-analytic synthesis. In addition, we performed subgroup analyses to compare the effects of hydrogel-assisted versus non-hydrogel delivery systems and to examine differences in therapeutic efficacy between exosomes derived from human and rat cells, aiming to provide refined insights into regenerative potential and translational optimization.

## Materials and methods

This meta-analysis was prospectively registered in PROSPERO (CRD420250654980) prior to initiation and was conducted in accordance with the Preferred Reporting Items for Systematic Reviews and Meta-Analyses (PRISMA) guidelines (Li et al., 2024).

### Search strategy

As of 15 August 2025, a systematic search was conducted in PubMed, Embase, and the Cochrane Library. The detailed search strategy is provided in Supplementary Material. In addition, a manual search of the reference lists of relevant studies was performed to identify additional eligible articles.

Eligibility criteria for this systematic review and meta-analysis were defined according to the PICOS framework. The population of interest comprised rat models of tail disc degeneration induced by repeated tail puncture and treated with SC-sEVs to evaluate therapeutic effects. Studies involving non-rat species, *in vitro* experiments, or other nonrelevant animal models were excluded. All types of SC-sEVs were considered, including xenogeneic and allogeneic sources, administered via local intradiscal injection with or without hydrogel loading. Eligible comparators included nonfunctional solutions (phosphate-buffered saline [PBS] or normal saline), hydrogels, or no treatment, while studies without appropriate experimental or control groups were excluded.

The outcomes of interest were MRI (magnetic resonance imaging) Pfirrmann grading, disc height index (DHI), and histological grading of intervertebral disc tissue (Xing et al., 2021; Li et al., 2024). Study characteristics (e.g., publication year, species, age, sex, and weight) were also extracted, and outcomes most consistently reported at 4 and 8 weeks were prioritized for analysis. Eligible studies were full-text, English-language randomized controlled trials (RCTs) of SC-sEVs versus PBS, saline, hydrogels, or no treatment in rat models of tail disc degeneration. Excluded studies included reviews, non-RCTs, commentaries, case reports/series, duplicates, *in vitro* studies, clinical trials, and those lacking sufficient data or clear exosome source.

### Data extraction and quality assessment

Two authors independently extracted study characteristics, population details, intervention protocols, study design, and outcomes (MRI Pfirrmann grading, DHI, and histological grading). Data extracted for outcomes included number of subjects, mean and standard deviation in either group. Risk of bias was independently assessed by two reviewers using the SYRCLE tool, which evaluates selection, performance, detection, attrition, reporting, and other biases. Any discrepancies in the aforementioned parts were resolved through discussion with another independent and senior author (the third author) until consensus was reached.

### Excerpt–outcomes assessment

The included studies evaluated IVDD using structural, imaging, and histological outcomes. MRI–based Pfirrmann grading was commonly employed to classify disc degeneration according to signal intensity and structural characteristics. In this system, grade 1 denotes a nucleus with a homogeneous bright appearance, grade 2 indicates inhomogeneous structure and clear distinction between the dark annulus and bright nucleus, grade 3 represents unclear distinction between nucleus and annulus, grade 4 represents lost distinction between nucleus and annulus, and normal to moderate disc height collapse, and grade 5 reflects advanced degeneration with collapsed disc space (Jin et al., 2024).

In addition, the DHI was calculated by referencing the vertebral midline, defined as a line connecting the centers of adjacent vertebral bodies. Both vertebral body height and intervertebral disc height were measured along this line, and the ratio of disc height to vertebral body height was expressed as the DHI (Hiraishi et al., 2018). A reduction in DHI was considered indicative of progressive degeneration. Beyond imaging measures, histological grading of intervertebral disc tissue was also reported as outcome indicators, providing microscopic and cellular-level evidence of IVDD severity.

### Statistical analysis

Mean differences (MD) of continuous outcomes were synthesized across studies using a random-effects model according to the DerSimonian and Laird method, in order to account for potential heterogeneity among individual studies. Statistical heterogeneity across studies was quantified using the I^2^ statistic, with values of 25%, 50%, and 75% considered low, moderate, and high heterogeneity, respectively. The effects of human- versus rat-derived exosomes were compared using a mixed-effects model, with subgroup (human vs. rat) specified as a fixed effect. Potential publication bias was examined visually with funnel plots and statistically using Egger’s intercept test, providing a formal assessment of small-study effects. A two-sided P value < 0.05 was considered statistically significant. The meta-analysis was conducted using Comprehensive Meta-Analysis, Version 3 (Englewood, NJ 2014).

## Results

### Studies eligible for analysis

**FIGURE 1:**
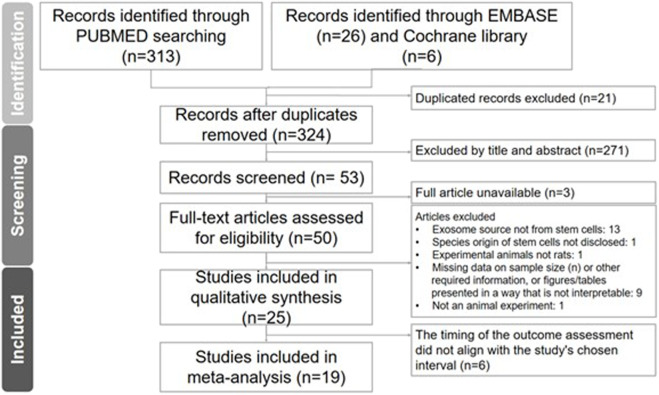
Literature search. Preferred reporting items for Systematic Reviews and Meta-Analysis (PRISMA) flow diagram illustrated the process for study inclusion.

Mesh terms were utilized to retrieve 313, 26, and 6 studies from PubMed, the Embase and the Cochrane library databases, respectively. After screening for duplicates and inconsistencies based on abstracts and titles, a total of 53 studies proceeded to the next round. Ultimately, a total of studies were included in the systematic review and of whom 19 articles (comprising 19 animal experiments involving 305 rats) were included in the meta-analysis (Figure 1; Table 1) (Ambrosio et al., 2024; Jin et al., 2024; Zhan et al., 2025; Hu et al., 2024; Shi et al., 2024; Xing et al., 2021; Chen et al., 2025; Ma et al., 2024; Zhang et al., 2024; Hu et al., 2023; Xu et al., 2023; Xiao et al., 2022; Guo et al., 2021; Liao et al., 2021; Sun et al., 2021; Liao et al., 2019; Guan et al., 2023; Liu et al., 2023; Liao et al., 2022).

**TABLE 1:** Characteristics of the experimental animals, including species, sex, age, relevance, and injection site.

Exosome delivery method	Study, year	Experimental animals	Sex, age	Site of experiment	The origin of exosomes	Frequency of injections	Method of verification
Non-hydrogel exosome	Chen et al. (2025)	SD rats, n = 6	Male, 8 weeks	Caudal vertebra	BMSC from femurs of 8wks male rat	Once a week	DHI (4 weeks), histological score (4 weeks)
	Ambrosio et al. (2024)	SD rats, n = 12	Male, 10–12 weeks	Co5/6; Co6/7; Co7/8	BMSC from human	Once	DHI (4 and 8 weeks)
	Jin et al. (2024)	SD rats, n = 4	Unspecified	Co7/8	BMSC from human	Once a week	DHI (4 and 8 weeks), MRI Pfirrmann (4 and 8 weeks)
	Ma et al. (2024)	SD rats, n = 5	Male, 8 weeks	Co8/9	BMSC from femurs of 8wks male rat	Once a week	Histological score (4 weeks), MRI Pfirrmann (4 weeks)
	Zhang et al. (2024)	SD rats, n = 15	Male, 3 months	Co6/7; Co7/8; Co8/9	BMSC from rats	Once a week	Histological score (4 weeks), MRI Pfirrmann (4 weeks)
	Hu et al. (2023)	SD rats, n = 3	Unspecified	Co5/6	UCMSC from human	Once	DHI (6 weeks), MRI Pfirrmann (6 weeks), histological score (6 weeks)
	Xu et al. (2023)	SD rats, n = 3	Female	Co7/8	BMSC from human	Once	Histological score (4 weeks), MRI Pfirrmann (4 weeks)
	Xiao et al. (2022)	SD rats, n = 6	Male	Co 9/10	BMSC from 6 to 8 weeks SD rat	Once	Histological score (8 weeks)
	Guo et al. (2021)	SD rats, n = 15	3 months	Co4/5, Co5/6, Co6/7	USC from male human	2nd and 4th weeks	DHI (4 and 8weeks), histological score (8 weeks)
	Liao et al. (2021)	SD rats, n = 6	Male, 6 weeks	Co8/9	BMSC from human	Once a week	DHI (4 weeks), histological score (4 weeks)
	Sun et al. (2021)	SD rats, n = 3	12 weeks	Co5/6	iPSCs-MSC from human	1st, 3rd, 5th, 7th weeks	Histological score (8 weeks), MRI Pfirrmann (8 weeks)
	Liao et al. (2019)	SD rats, n = 20	3 months	Co7/8, Co8/9, Co9/10	BMSC from human	Every 2 weeks	DHI (4 and 8 weeks), MRI Pfirrmann (4 and 8 weeks), histological score (8 weeks)
The experimental group included both hydrogel and non-hydrogel	Guan et al. (2023)	Rats, n = 3	12 weeks	Co8/9	BMSC from femurs of SD rats	Once	Histological score (8 weeks), MRI Pfirrmann (8 weeks)
	Liu et al. (2023)	SD rats, n = 4	Unspecified	Co7/8	BMSC from femurs and tibias of 4weeks rat	Once	DHI (8 weeks), MRI Pfirrmann (8 weeks)
	Liao et al. (2022)	SD rats, n = 6	Male, 8 weeks	Co7/8, Co8/9	BMSC from human	Once a week	DHI (4 and 8 weeks), MRI Pfirrmann (4 and 8 weeks), histological score (8 weeks)
Hydrogel exosome	Zhan et al. (2025)	SD rats, n = 5	Male, 12 weeks	Co5/6	iPSCs-MSC from human	The first and 5th weeks	Histological score (8 weeks)
	Hu et al. (2024)	SD rats, n = 3	Male	Co3/4	BMSC from rats	Once	DHI (4 weeks)
	Shi et al. (2024)	SD rats, n = 4	Unspecified	Co6/7	BMSC from femur of 2–3 months rat	Once a week	MRI Pfirrmann (6 weeks)
	Xing et al. (2021)	SD rats, n = 3	Male	Co7/8, Co8/9, Co9/10	ADSC from rats	Once	DHI (4 weeks)

### Overall effect at 4–8 weeks

**FIGURE 2:**
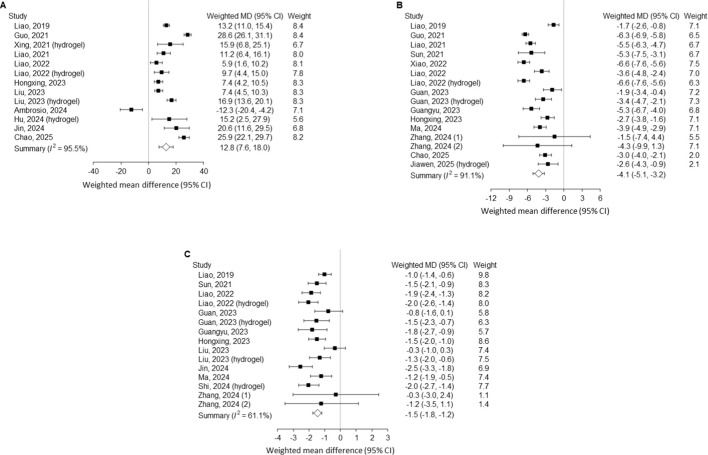
Outcomes at 4–8 weeks after needle puncture–induced disc injury comparing exosome treatment with controls. **(A)** Disc height index; **(B)** Histological score; **(C)** MRI Pfirrmann grading. MD, mean difference; CI, confidence interval; MRI, magnetic resonance imaging.

Meta-analysis of studies evaluating outcomes 4–8 weeks after needle-puncture–induced intervertebral disc injury demonstrated significant therapeutic effects of exosome treatment compared with controls (Figures 2A–C). When multiple time points were available within this interval, data nearest to 4 weeks were selected for pooled analysis. Exosome administration significantly improved the DHI (weighted MD = 12.8%, 95% CI 7.6–18.0), reduced histological scores (weighted MD = −4.1, 95% CI −5.1 to −3.2), and decreased MRI signal intensity (weighted MD = −1.5, 95% CI −1.8 to −1.2). Heterogeneity across studies was considerable for DHI and histological score (I^2^ = 95.5% and 91.1%, respectively) and moderate for MRI signal intensity (I^2^ = 61.1%). Overall, these findings indicate that exosome therapy confers early structural and morphological restoration of the intervertebral disc following injury.

### Time-point analysis

**FIGURE 3:**
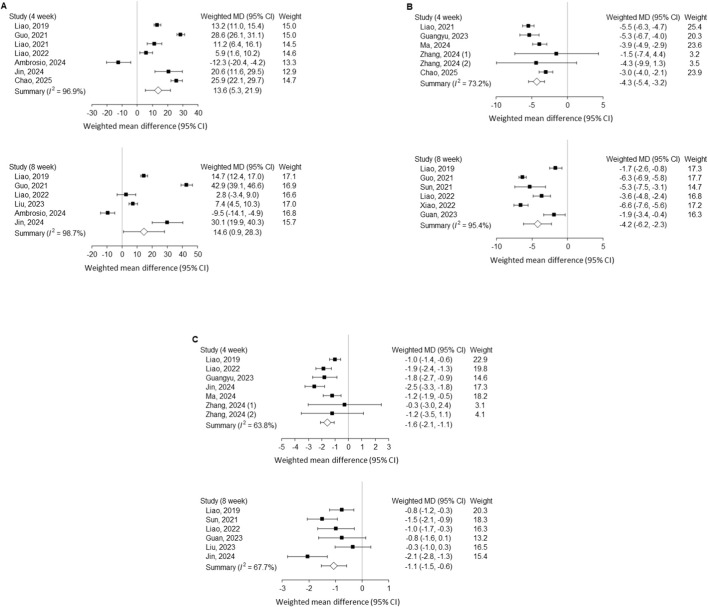
Outcomes after needle puncture–induced disc injury comparing exosome treatment with controls, stratified by 4 and 8 weeks. **(A)** Disc height index; **(B)** Histological score; **(C)** MRI Pfirrmann grading. MD, mean difference; CI, confidence interval; MRI, magnetic resonance imaging.

Stratified analyses by time point further demonstrated consistent therapeutic efficacy of non-hydrogel exosome treatment at both 4 and 8 weeks after disc injury (Figures 3A–C). Specifically, the subgroup analysis limited to studies using non-hydrogel exosomes revealed significant improvements at 4 weeks in disc height index (DHI; weighted MD = 13.6%, 95% CI 5.3–21.9), histological scores (weighted MD = −4.3, 95% CI −5.4 to −3.2), and MRI signal intensity (weighted MD = −1.6, 95% CI −2.1 to −1.1). At 8 weeks, exosome therapy continued to exert comparable benefits, with improvements in DHI, histological scores, and MRI findings consistent with those observed at 4 weeks. Substantial heterogeneity was noted across these outcomes (I^2^ > 50%). Collectively, these results indicate that non-hydrogel exosome administration provides both early and sustained protective effects against disc degeneration.

### Hydrogel-assisted delivery

Analysis of studies employing hydrogel-based delivery systems (Supplementary Figures S1A–C, S2A–C) demonstrated that combining exosomes with hydrogels further enhanced therapeutic efficacy. Compared with PBS, the exosome–hydrogel combination produced greater improvement in DHI (weighted MD = 14.4%, 95% CI: 10.2–18.5). Moreover, compared with hydrogel alone, the combination significantly reduced histological scores (weighted MD = −4.2, 95% CI: −6.8 to −1.7). These results indicate that hydrogel-based delivery prolongs exosome retention and augments therapeutic benefits.

### Subgroup analysis by delivery vehicle

**FIGURE 4:**
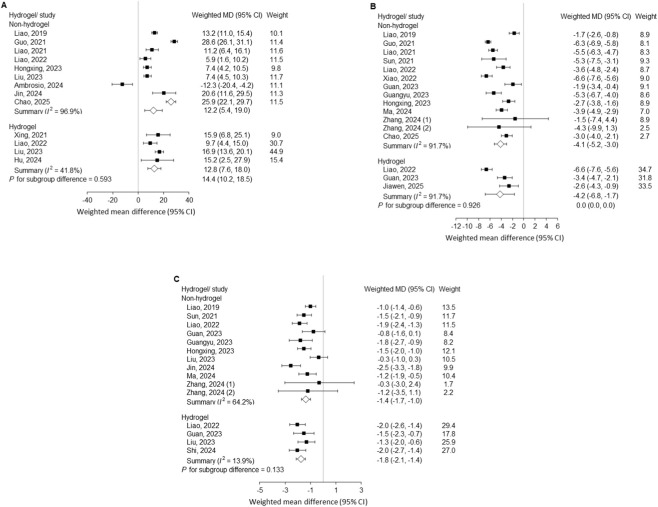
Outcomes after needle puncture–induced disc injury comparing exosome treatment with controls, stratified by delivery method (hydrogel-based vs. non-hydrogel). Subgroup analyses with *P* for subgroup difference are shown for **(A)** Disc height index; **(B)** Histological score; and **(C)** MRI Pfirrmann grading. MD, mean difference; CI, confidence interval; MRI, magnetic resonance imaging.

Subgroup analyses comparing hydrogel-based versus non-hydrogel exosome delivery demonstrated similar therapeutic efficacy across all measured outcomes (Figures 4A–C). In the non-hydrogel subgroup, exosome treatment significantly improved DHI (weighted MD = 12.2%, 95% CI 5.4–19.0), reduced histological scores (weighted MD = −4.1, 95% CI −5.2 to −3.0), and decreased MRI signal intensity (weighted MD = −1.4, 95% CI −1.7 to −1.0). Comparable benefits were observed in the hydrogel subgroup, with DHI improvement (weighted MD = 14.4%, 95% CI 10.2–18.5), reduction in histological scores (weighted MD = −4.2, 95% CI −6.8 to −1.7), and MRI attenuation (weighted MD = −1.8, 95% CI −2.1 to −1.4). Between-group comparisons revealed no statistically significant differences in treatment effect for any outcome (DHI, p = 0.593; histology, p = 0.926; MRI, p = 0.133). Although heterogeneity remained high in the non-hydrogel subgroup (I^2^ > 60%), it was markedly reduced in hydrogel studies, particularly for MRI (I^2^ = 13.9%). These findings suggest that while both delivery strategies confer comparable regenerative efficacy, hydrogel encapsulation may provide more consistent outcomes with reduced inter-study variability.

### Subgroup analysis by exosome source

**FIGURE 5:**
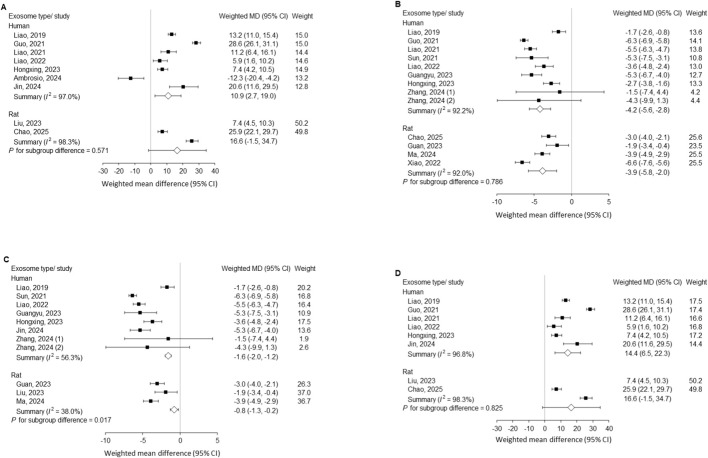
Outcomes after needle puncture–induced disc injury comparing exosome treatment with controls, stratified by exosome source (rat vs. human). Subgroup analyses with P for subgroup difference are shown for **(A)** Disc height index; **(B)** Histological score; **(C)** MRI Pfirrmann grading; and **(D)** Sensitivity analysis for the DHI, demonstrating result stability after exclusion of the highly heterogeneous study by Ambrosio et al. (2024). MD, mean difference; CI, confidence interval; MRI, magnetic resonance imaging.

Both human- and rat-derived exosomes improved disc height index and histological scores, with no significant subgroup differences (P = 0.571 and 0.786, respectively). In contrast, for MRI signal intensity, both subgroups showed significant improvement compared with controls, but human-derived exosomes exhibited slightly greater effects (weighted MD, −1.6; 95% CI, −2.0 to −1.2) than rat-derived exosomes (weighted MD, −0.8; 95% CI, −1.3 to −0.2), with a significant subgroup difference (P = 0.017) (Figures 5A–C).

### Sensitivity analyses

Sensitivity analyses demonstrated that the therapeutic effects of exosome treatment on DHI and histological outcomes were robust (Figure 5D).

For DHI at 4 weeks, exclusion of the studies by Guo et al. (2021), Ambrosio et al. (2024), and Chen et al. (2025) reduced heterogeneity from 96.9% to 75.7%, while the treatment effect remained significant (MD 11.8, 95% CI 7.3–16.3; Supplementary Figure S5A). At 8 weeks, treatment benefits persisted; however, heterogeneity remained high despite conducting leave-one-out analysis (I^2^ 98.7%). For histological scores, removal of Chen et al. (2025) at the 4-week timepoint decreased I square to 68.6%, with preserved statistical significance (MD −4.4, 95% CI −5.5 to −3.3; Supplementary Figure S5B). At 8 weeks, exclusion of Guo et al. (2021) and Xiao et al. (2022) reduced I square to 78.3%, again without loss of treatment effect (MD −2.9, 95% CI −4.4 to −1.5; Supplementary Figure S5C). These variations likely reflect differences in study design, such as the use of urine-derived stem cell EVs instead of BMSC-derived EVs in Guo et al. (2021), and the more aggressive disc injury protocol applied by Ambrosio et al. (2024), who used an 18-gauge needle across three discs levels. Meta-regression incorporating puncture needle size and injury level did not reveal significant associations with DHI, histological scores, or MRI Pfirrmann grades at either 4 or 8 weeks. Other potentially relevant moderators, including EV dose, concentration, and stem cell origin, could not be evaluated due to insufficient reporting. Despite these limitations, the persistence of significant treatment effects following sensitivity analyses supports the conclusion that the therapeutic benefits of exosome therapy are consistent and robust, even in the presence of moderate residual heterogeneity.

### Methodological quality and risk of bias

Out of the 19 included studies, all reported random sequence generation, and 9 of them explicitly described randomization procedures, indicating a low risk of selection bias. However, allocation concealment and blinding of participants or personnel were insufficiently detailed in most reports, resulting in an unclear risk of performance bias. Likewise, outcome assessment blinding was generally unreported. All studies provided complete outcome data and demonstrated low risks for attrition and selective reporting biases. No evidence of other potential biases was identified. Overall, the methodological quality of most studies was deemed acceptable and consistent with reliable experimental standards (Supplementary Figure S3).

### Publication bias

Funnel plots of the standard error versus mean differences in disc height index, histological scores, and MRI signal intensity were generated (Supplementary Figures S4A–C). The plots appeared symmetrical, and Egger’s test indicated no evidence of publication bias (P = 0.423, 0.219, and 0.966, respectively).

## Discussion

This meta-analysis demonstrated that EVs therapy significantly alleviated structural and histological degeneration in experimental models of IVDD, as evidenced by improvements in disc height index, histological grading, and MRI Pfirrmann scores at both 4 and 8 weeks. The consistent findings across time points suggest a sustained regenerative effect of EVs on disc morphology and cellular integrity.

Subgroup analyses provided further insight into factors modulating therapeutic efficacy. In the comparison between hydrogel-based and non-hydrogel exosome delivery systems, both approaches demonstrated significant improvements in disc height, histological architecture, and MRI signal intensity relative to controls, with no statistically significant differences between groups. These results indicate that exosome treatment exerts robust regenerative effects regardless of the delivery vehicle. However, studies employing hydrogel scaffolds exhibited lower heterogeneity and narrower confidence intervals, suggesting that hydrogels may enhance result consistency by stabilizing vesicular structure, promoting sustained release, and improving local retention of EVs within the avascular disc microenvironment.

Furthermore, species-based subgroup analyses revealed comparable regenerative effects between human- and rat-derived exosomes in terms of disc height index and histological scores, with no significant subgroup differences (P = 0.571 and P = 0.786, respectively). In contrast, for MRI signal intensity, both subgroups showed significant improvement compared with controls; however, human-derived exosomes exhibited a slightly greater effect (weighted MD = −1.6, 95% CI –2.0 to −1.2) than rat-derived exosomes (weighted MD = −0.8, 95% CI –1.3 to −0.2), with a statistically significant subgroup difference (P = 0.017). The superior performance of human-derived EVs in MRI outcomes, despite the xenogeneic barrier, may be attributed to the inherent “immune privilege” of MSC-derived sEVs. These vesicles typically express low levels of major histocompatibility complex (MHC) class I and lack MHC class II molecules, thereby minimizing host immune rejection even across species lines (Kumar et al., 2024). Mechanistically, human MSCs may secrete a more potent repertoire of anti-inflammatory miRNAs and trophic factors compared to rodent MSCs, potentially leading to more robust matrix synthesis and water retention reflected in MRI signals. However, potential immunogenicity remains a concern for repeated long-term dosing, and future studies should specifically monitor host immune responses (e.g., antibody production) against xenogeneic EV surface proteins. These findings suggest that while both sources of exosomes confer structural and histological benefits, species-specific molecular profiles and intercellular signaling mechanisms may influence their ability to modulate disc hydration and MRI-detectable tissue restoration.

Collectively, these results support the therapeutic promise of EVs for IVDD and underscore the need to refine both their delivery strategy and cellular origin to optimize translational outcomes.

EVs play a multifaceted role in preserving the extracellular matrix microenvironment and maintaining nucleus pulposus cell homeostasis through the transfer of proteins, transcription factors, and regulatory RNAs (Kumar et al., 2024). By mediating intercellular communication, EVs can modulate inflammation, apoptosis, and extracellular matrix remodeling, thereby delaying degenerative processes within the disc (Liu and Wang, 2023). As an acellular regenerative approach, EV therapy provides comparable biological benefits to stem-cell transplantation while avoiding the risks of immune rejection, or tumorigenicity (Joshi et al., 2025; Chen et al., 2024). Compared with previous meta-analyses that included studies up to 2022 (Li et al., 2024), the present study expanded the search to encompass publications through 2025, integrating 19 datasets restricted to rat models to minimize interspecies variability and improve statistical consistency. Furthermore, subgroup analyses were conducted based on hydrogel versus non-hydrogel delivery systems and on the cellular origin of EVs (rat- or human-derived). These stratified findings not only strengthen the evidence supporting EV efficacy but also highlight that both the delivery microenvironment and vesicle source are critical determinants of therapeutic outcome.

Subgroup analysis by cellular origin showed that both human- and rat-derived EVs improved disc height index and histological scores to a similar extent, with no significant subgroup differences (DHI: P = 0.571; histology: P = 0.786). For MRI signal intensity, both subgroups improved versus controls; however, human-derived EVs produced a modestly greater effect (weighted MD = −1.6, 95% CI −2.0 to −1.2) than rat-derived EVs (weighted MD = −0.8, 95% CI −1.3 to −0.2), yielding a significant subgroup difference (P = 0.017). These findings suggest broadly comparable structural and histological benefits across EV sources, with a potential MRI-level advantage for human-derived preparations. Given the limited number of rat-EV studies and substantial heterogeneity, this difference should be interpreted cautiously and validated in adequately powered, head-to-head experiments.

Additional analyses examining the use of hydrogel carriers provided further insight into the importance of delivery strategies in EV-based disc regeneration. Compared with normal saline controls, hydrogel-encapsulated EVs produced significant improvements in disc height index, histological architecture, and MRI grading (Supplementary Figures S1A–C), confirming their therapeutic efficacy. When compared with hydrogel scaffolds alone, the EV-loaded constructs continued to demonstrate superior outcomes, indicating that the regenerative benefit was mainly derived from the biological activity of EVs rather than from the scaffold material itself. The incorporation of EVs into a hydrogel matrix offers several advantages: it protects vesicles from enzymatic degradation, enables sustained and localized release, and maintains a hydrated three-dimensional environment conducive to extracellular matrix remodeling and cell survival (Ju et al., 2022). These attributes are particularly relevant for the avascular and nutrient-limited intervertebral disc, where prolonged bioavailability of EVs is crucial for therapeutic success. Collectively, these findings support the concept that hydrogel-based delivery systems can serve as a promising platform to enhance the stability, retention, and functional efficacy of EVs in IVDD treatment.

Despite the strengthened methodological rigor and expanded dataset in the present meta-analysis, several limitations should be acknowledged. First, although the inclusion of studies up to 2025 increased the statistical power compared with previous reviews, variability in experimental design remains a major concern. The dosing regimen, timing of EV administration, and concentration of injected vesicles were not standardized across studies, which may have introduced heterogeneity in the pooled estimates. Second, for the outcome of DHI, several studies reported values that markedly deviated from other datasets, likely due to differences in measurement units or normalization methods (Ma et al., 2024; Zhang et al., 2024; Xu et al., 2023; Guan et al., 2023). However, as these studies did not specify the exact units or calculation criteria, conversion to a standardized scale was not feasible; therefore, their DHI data were excluded from the pooled quantitative synthesis to avoid distortion of the overall effect size.

Notably, the unexpectedly greater DHI reduction in the BM-MSC-EV group compared with the sham group during the first eight weeks of the study by Ambrosio et al. contrasts with most reports, which show protective effects of stem-cell-derived EVs. This paradoxical trend may be related to the model induction procedure, as the authors used a relatively large 18-gauge needle to puncture the disc, causing more severe annular and endplate disruption. Such extensive mechanical injury could have overwhelmed the early reparative potential of BM-MSC-EVs, further contributing to inter-study variability in DHI trajectories.

In addition, information regarding the sex of the animals, the specific coccygeal levels injected, and the source and passage of the parent stem cells used for EV production was inconsistently reported, further limiting the comparability of results. A critical, often overlooked limitation is the batch-to-batch variability inherent in biologic products. Differences in donor age, tissue source (e.g., adipose vs. bone marrow), and cell culture passage number can significantly alter the cargo and therapeutic potency of sEVs (Witwer et al., 2019). For instance, sEVs derived from late-passage MSCs often show reduced regenerative capacity compared to early-passage counterparts. The lack of standardized “potency assays” in the included studies makes it difficult to normalize the biological dose across different experiments, contributing to the observed heterogeneity. Moreover, all included experiments were conducted in rat models, and while this restriction minimized interspecies variability, it also limits the direct extrapolation of findings to human clinical scenarios. Finally, data extraction from published figures may have introduced minor quantitative errors despite the use of standardized software. Collectively, these limitations underscore the need for rigorous standardization in future research. To facilitate clinical translation, researchers should adhere to the minimal information for studies of extracellular vesicles (MISEV) guidelines (Théry et al., 2018) to ensure consistent characterization. Furthermore, future studies must move beyond single-dose efficacy proofs to establish clear dose-response relationships and determine optimal therapeutic windows. Long-term follow-up (>3 months) is also essential to verify whether the structural benefits observed on MRI translate into sustained biomechanical restoration and pain relief.

## Conclusion

This systematic review and meta-analysis demonstrated that SC-sEVs significantly attenuate intervertebral disc degeneration in rat models, as evidenced by improvements in disc height, histological architecture, and MRI Pfirrmann grading. The beneficial effects were consistently observed at both short- and mid-term follow-up, supporting the sustained regenerative potential of EVs. Subgroup analyses further revealed that both hydrogel-based and non-hydrogel delivery systems achieved comparable therapeutic outcomes, with hydrogel scaffolds exhibiting reduced heterogeneity and more consistent results, likely owing to improved local retention and sustained release. Similarly, both human- and rat-derived EVs exerted significant regenerative effects, with a modest MRI advantage observed for human-derived preparations, suggesting that cross-species biological activity is largely preserved. Collectively, these findings highlight EVs as promising acellular therapeutics capable of modulating inflammation, reducing apoptosis, and promoting extracellular matrix repair in degenerative discs. Nevertheless, the variability in EV sources, dosing, and delivery strategies underscores the need for standardized experimental protocols and translational studies aimed at optimizing EV characterization, dosage, and delivery for clinical application in intervertebral disc regeneration.

## Data Availability

The original contributions presented in the study are included in the article/Supplementary Material, further inquiries can be directed to the corresponding author.

## Supplementary material

SUPPLEMENTARY FIGURE S1 Outcomes after needle puncture–induced disc injury comparing exosome–hydrogel treatment with controls. **(A)** Disc height index; **(B)** Histological score; **(C)** MRI Pfirrmann grading. MD, mean difference; CI, confidence interval; MRI, magnetic resonance imaging.

SUPPLEMENTARY FIGURE S2 Outcomes after needle puncture–induced disc injury comparing exosome–hydrogel treatment with hydrogel alone. **(A)** Disc height index; **(B)** Histological score; **(C)** MRI Pfirrmann grading. MD, mean difference; CI, confidence interval; MRI, magnetic resonance imaging.

SUPPLEMENTARY FIGURE S3 Summary of risk of bias.

SUPPLEMENTARY FIGURE S4 The assessment of publication bias for **(A)** Disc height index; **(B)** Histological score; **(C)** MRI Pfirrmann grading. MRI, magnetic resonance imaging.

SUPPLEMENTARY FIGURE S5 Sensitivity analysis for the **(A)** Disc height index, 4 weeks; **(B)** Histological score, 4 weeks; **(C)** Histological score, 8 weeks, demonstrating result stability after exclusion of the highly heterogeneous study.
