# Supplementary figures and images for: Therapeutic effects of stem cell–derived extracellular vesicles in animal models of intervertebral disc degeneration: a systematic review and meta-analysis of species differences and delivery strategies
Source: Front Bioeng Biotechnol. 2026 Jan 30;14:1749916. doi: 10.3389/fbioe.2026.1749916 (PMC12901408; doi:10.3389/fbioe.2026.1749916)

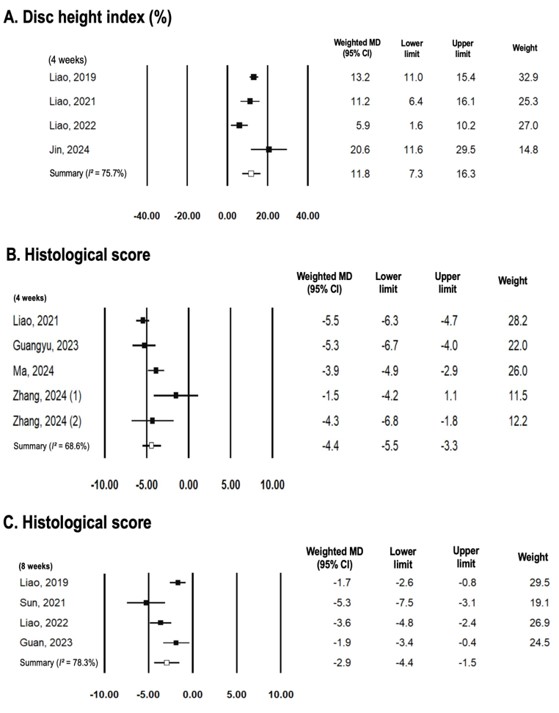

Supplement: Supplementary file 1 [file Image5.jpg]

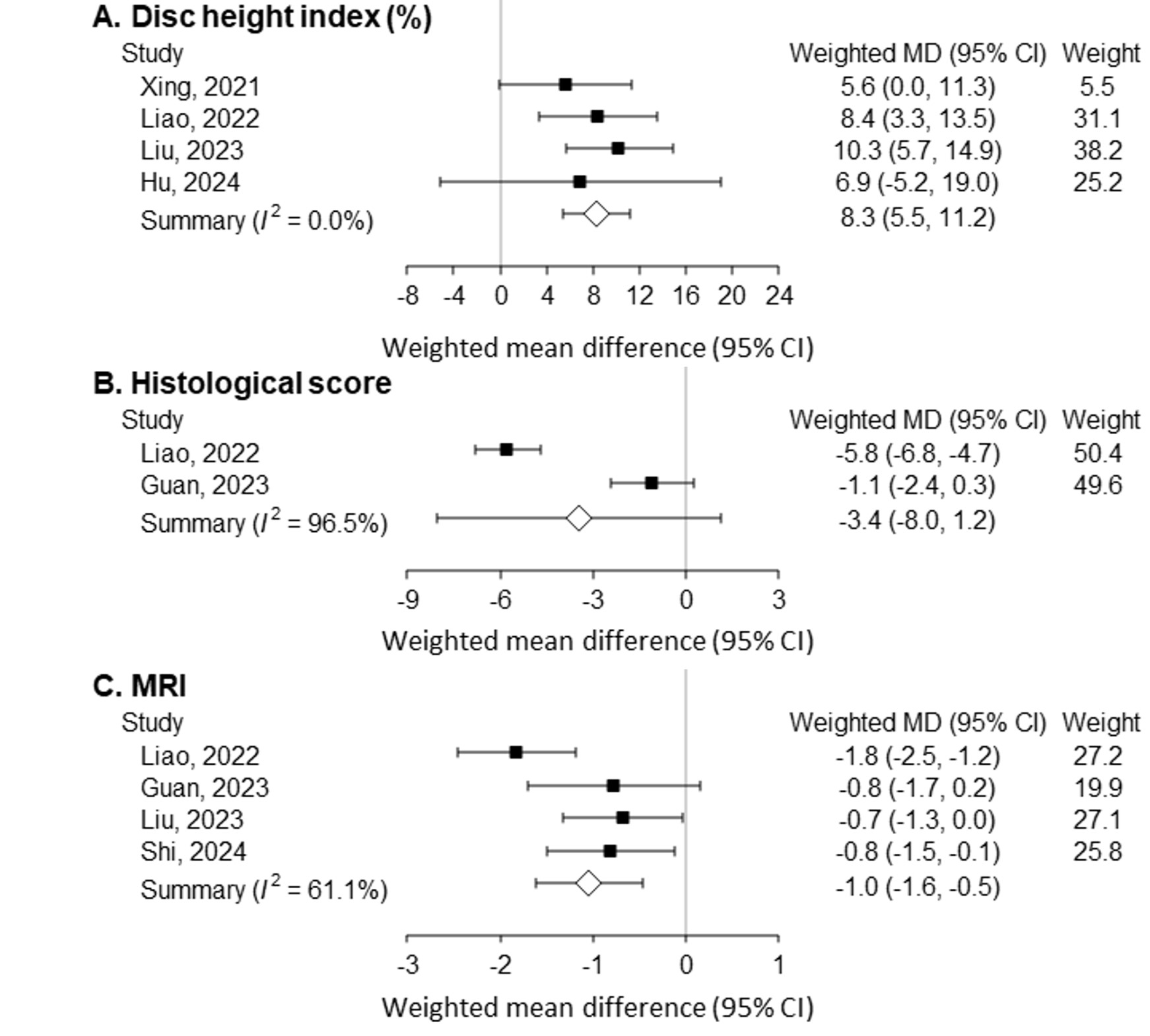

Supplement: Supplementary file 2 [file Image2.jpg]

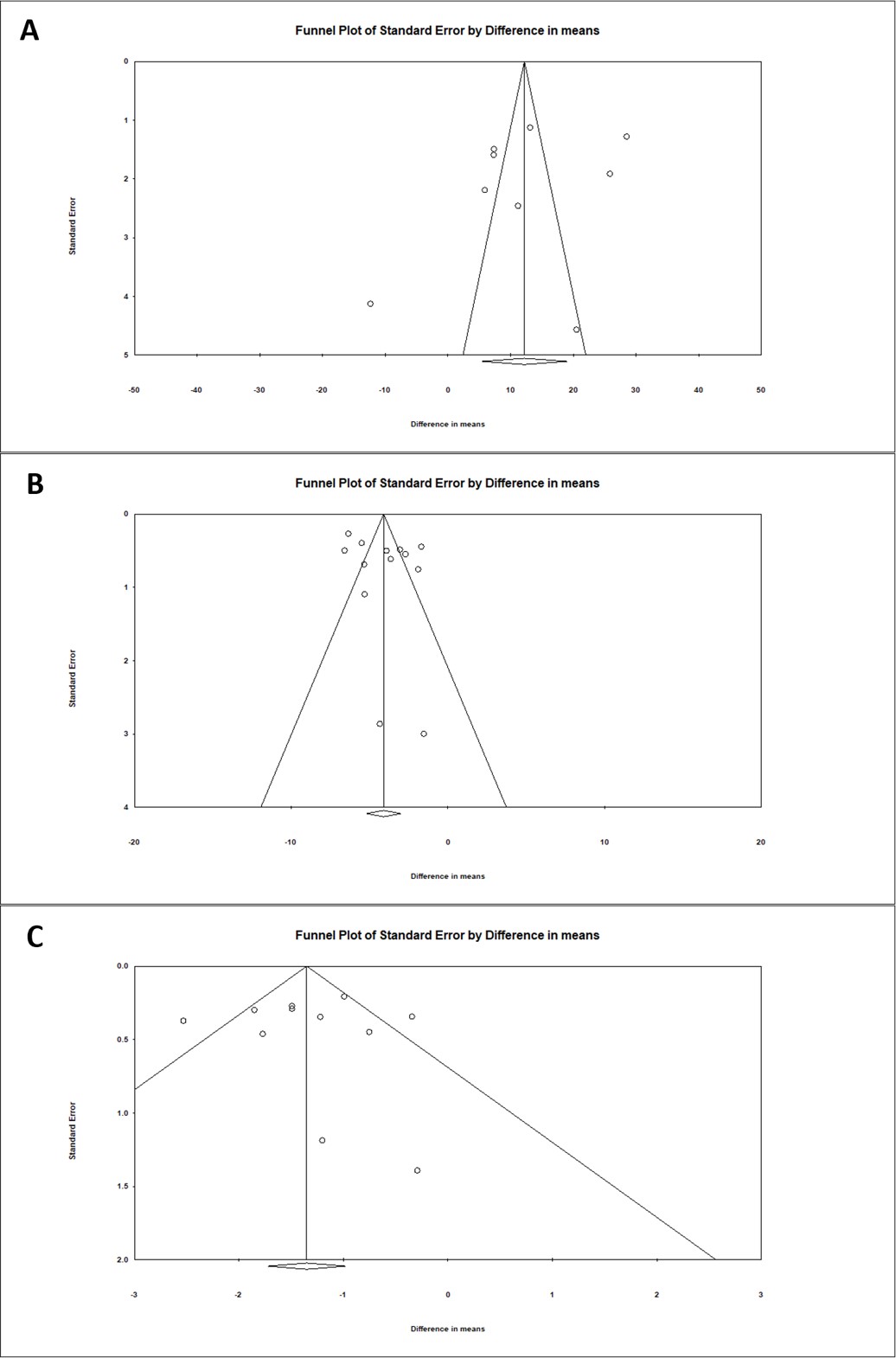

Supplement: Supplementary file 6 [file Image4.jpg]

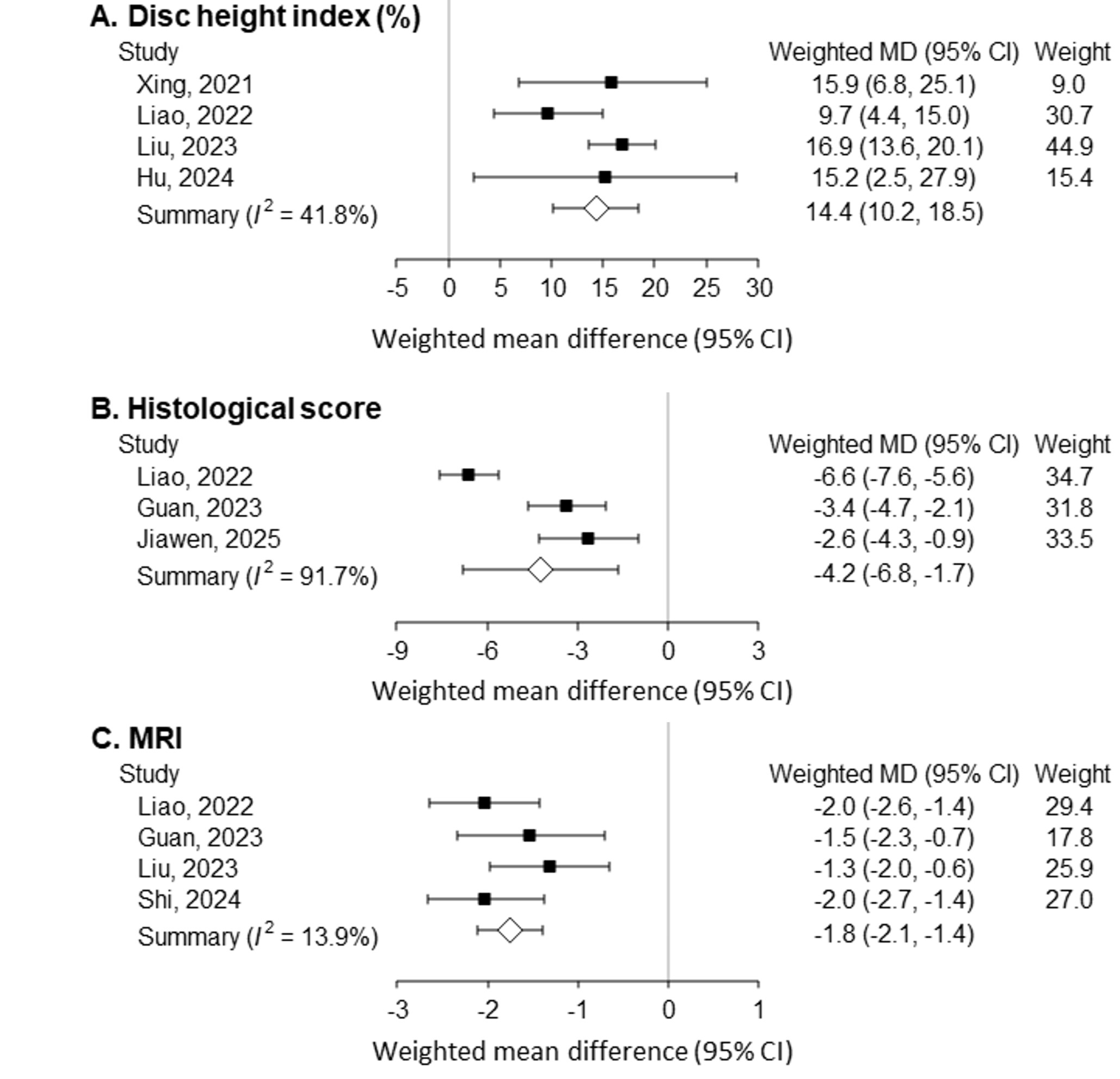

Supplement: Supplementary file 7 [file Image1.jpg]
