# Supplementary material for: Therapeutic effects of stem cell–derived extracellular vesicles in animal models of intervertebral disc degeneration: a systematic review and meta-analysis of species differences and delivery strategies
Source: Front Bioeng Biotechnol. 2026 Jan 30;14:1749916. doi: 10.3389/fbioe.2026.1749916 (PMC12901408; doi:10.3389/fbioe.2026.1749916)
Supplement: Supplementary file 3 [file Image3.pdf]

	Random sequence generation (selection bias)	Allocation concealment (selection bias)	Blinding of participants and personnel (performance bias)	Blinding of outcome assessment (detection bias)	Incomplete outcome data (attrition bias)	Selective reporting (reporting bias)	Other bias
Ambrosio L 2024	+	?	?	?	+	+	+
Chao Chen 2025	+	?	?	?	+	+	+
Guangyu Xu 2023	+	?	?	?	+	+	?
Guan M 2023	+	?	?	?	+	+	?
Guo Z 2021	?	?	?	?	+	+	+
Hongxing Hu 2023	?	?	?	?	+	+	+
Hu S 2024	?	?	?	?	+	+	+
Jiawen Zhan 2025	+	?	?	?	+	+	+
Jin Y 2024	?	?	?	?	+	+	+
Liao Z 2019	?	?	?	?	+	+	+
Liao Z 2021	+	?	?	?	+	+	+
Liao Z 2022	?	?	?	?	+	+	+
Liu C 2023	+	?	?	?	+	+	+
Ma S 2024	+	?	?	?	+	+	?
Shi P 2024	+	?	?	?	+	+	+
Sun Y 2021	?	?	?	?	+	+	+
Xiao Q 2022	?	?	?	?	+	+	+
Xing H 2021	?	?	?	?	+	+	+
Zhang W 2024	?	?	?	?	+	+	?
