# Supplementary material for: Therapeutic effects of stem cell–derived extracellular vesicles in animal models of intervertebral disc degeneration: a systematic review and meta-analysis of species differences and delivery strategies
Source: Front Bioeng Biotechnol. 2026 Jan 30;14:1749916. doi: 10.3389/fbioe.2026.1749916 (PMC12901408; doi:10.3389/fbioe.2026.1749916)
Supplement: Supplementary file 4 [file Table1.doc]

| **Pubmed** |
| --- |
| 1. "exosomes"[MeSH Terms] OR "exosome*"[Title/Abstract] OR "nanoparticle*"[Title/Abstract] OR "microvesicle*"[Title/Abstract] OR "nanovesicle*"[Title/Abstract] OR "microparticle*"[Title/Abstract] OR "exovesicle*"[Title/Abstract] OR "extracellular vesicle*"[Title/Abstract] |
| 2. "Intervertebral disc"[MeSH Terms] OR "disc degeneration"[All Fields] OR "intervertebral disc*"[All Fields] OR "IVDD"[All Fields] OR "rat tail*"[All Fields] OR "rat tail*"[All Fields] OR "caudal disc*"[All Fields] |
| 3. "brain*"[Title/Abstract] OR "kidney"[Title/Abstract] OR "renal"[Title/Abstract] OR "heart*"[Title/Abstract] OR "spinal cord"[Title/Abstract] OR "stroke*"[Title/Abstract] OR "pancreas*"[Title/Abstract] OR "Alzheimer's disease"[Title/Abstract] OR "ovary"[Title/Abstract] OR "ovarian"[Title/Abstract] OR "diabet*"[Title/Abstract] OR "cancer*"[Title/Abstract] OR "neoplasm"[Title/Abstract] OR "tumor*"[Title/Abstract] |
| 1 AND 2 NOT 3 |
| **Embase** |
| 1. 'exosome'/exp |
| 2. 'exosome*':ab,ti OR 'microparticle*':ab,ti OR microvesicle*:ab,ti OR 'nanoparticle*':ab,ti OR 'nanovesicle*':ab,ti OR exovesicle*:ab,ti OR 'extracellular vesicle*':ab,ti |
| 3. 'intervertebral disk'/exp |
| 4. 'intervertebral disk*' OR 'disc degeneration':ab,ti OR ivdd:ab,ti OR 'rat tail*':ab,ti OR 'caudal disc*':ab,ti |
| 5. 'brain':ab,ti OR 'kidney':ab,ti OR 'renal':ab,ti OR heart:ab,ti OR 'spinal cord':ab,ti OR stroke:ab,ti OR 'pancreas':ab,ti OR 'alzheimers disease':ab,ti OR ovary:ab,ti OR ovarian:ab,ti OR diabet:ab,ti OR cancer:ab,ti OR 'neoplasm':ab,ti OR tumor:ab,ti |
| (1or 2) AND (3or4) NOT 5 |
| Filter: AND ('biomaterial'/dd OR 'endogenous compound'/dd OR 'nanoparticle'/dd) AND ('cell degeneration'/dm OR 'discogenic pain'/dm OR 'intervertebral disk degeneration'/dm OR 'intervertebral disk disease'/dm OR 'intervertebral disk hernia'/dm OR 'low back pain'/dm) AND ('animal experiment'/de OR 'animal model'/de OR 'in vivo study'/de OR 'rat model'/de) AND 'article'/it |
| **Cochrane library** |
| 1. MeSH descriptor: [Exosomes] explode all trees |
| 2. (exosome*):ti,ab,kw OR (nanoparticle*):ti,ab,kw OR (microvesicle*):ti,ab,kw OR (nanovesicle*):ti,ab,kw OR (microparticle*):ti,ab,kw |
| 3. (exovesicle*):ti,ab,kw OR (extracellular vesicle*):ti,ab,kw |
| 4. MeSH descriptor: [Intervertebral Disc] explode all trees |
| 5. (Intervertebral disc):ti,ab,kw OR (disc degeneration):ti,ab,kw OR (IVDD):ti,ab,kw OR (rat tail*):ti,ab,kw OR (rat-tail*):ti,ab,kw |
| 6. (caudal disc*):ti,ab,kw |
| (1or2or3) AND (4or5or6) |
